# Photodynamic Activity of Tribenzoporphyrazines with Bulky Periphery against Wound Bacteria

**DOI:** 10.3390/ijms21176145

**Published:** 2020-08-26

**Authors:** Magdalena Stolarska, Arleta Glowacka-Sobotta, Dariusz T. Mlynarczyk, Jolanta Dlugaszewska, Tomasz Goslinski, Jadwiga Mielcarek, Lukasz Sobotta

**Affiliations:** 1 Chair and Department of Inorganic and Analytical Chemistry, Poznan University of Medical Sciences, Grunwaldzka 6, 60-780 Poznan, Poland; magdalena.pawelek@gmail.com (M.S.); jmielcar@ump.edu.pl (J.M.); 2 Chair and Department of Maxillofacial Orthopedics and Orthodontics, Poznan University of Medical Sciences, Bukowska 70, 60-812 Poznan, Poland; aglow@ump.edu.pl; 3 Chair and Department of Chemical Technology of Drugs, Poznan University of Medical Sciences, Grunwaldzka 6, 60-780 Poznan, Poland; mlynarczykd@ump.edu.pl (D.T.M.); tomasz.goslinski@ump.edu.pl (T.G.); 4 Chair and Department of Genetics and Pharmaceutical Microbiology, Poznan University of Medical Sciences, Swiecickiego 4, 60-781 Poznan, Poland; jdlugasz@ump.edu.pl

**Keywords:** porphyrazins, singlet oxygen, photodynamic antimicrobial therapy, wound

## Abstract

Magnesium(II) tribenzoporphyrazines with phenoxybutylsulfanyl substituents were evaluated as photosensitizers in terms of their optical properties against wound bacteria. In the UV-vis spectra of analyzed tribenzoporphyrazines, typical absorption ranges were found. However, the emission properties were very weak, with fluorescence quantum yields in the range of only 0.002–0.051. What is important, they revealed moderate abilities to form singlet oxygen with the quantum yields up to 0.27. Under irradiation, the macrocycles decomposed via photobleaching mechanism with the quantum yields up to 8.64 × 10^−5^. The photokilling potential of tribenzoporphyrazines was assessed against *Streptococcus pyogenes*, *Staphylococcus epidermidis*, as well as various strains of *Staphylococcus aureus*, including methicillin-sensitive and-resistant bacteria. Both evaluated photosensitizers revealed high photodynamic potential against studied bacteria (>3 logs). *S.* *aureus* growth was reduced by over 5.9 log, methicillin-resistant *S. aureus* by 5.1 log, *S.* *epidermidis* by over 5.7 log, and *S. pyogenes* by over 4.7 log.

## 1. Introduction

A wound is described as a break in the integrity and continuity of a tissue, mucus membrane, or an organ tissue [1]. After an injury, wounds heal themselves according to physiological mechanisms. However, chronic wounds can appear when the mechanism is impaired in elderly patients and patients suffering from diabetes, malnutrition, or cancer, where the immune system is not as efficient as usual [1,2]. Skin is an essential anatomical barrier that offers protection from pathogens. Therefore, open wounds are susceptible to the microbial environment and, as a consequence, potential microbial infections. Four phases of wound healing can be distinguished: hemostasis, inflammatory phase, proliferative, and remodeling [1,2,3]. These stages, while occurring consecutively, do have a partial overlap [4]. The inflammatory phase begins directly after the injury, and it aims to stop the spread of the lesion and contain the damage to a confined area [2,4]. During this stage, the neutrophils, monocytes, and later macrophages, regulated by cytokines, clean the wound on a tissue level to dispose of any foreign material, including microorganisms. Next, in the proliferative phase, collagen and other components of connective tissue extracellular matrix are produced, along with the rapid division of the cells and angiogenesis. The final phase, which takes place up to several weeks after the injury, is based on the modification of the newly-formed scar tissue, which takes over the functions of the tissue which existed before the trauma.

The most common infections found in lesions are caused by *Staphylococcus aureus*, including methicillin-resistant strains (MRSA), *Streptococcus pyogenes*, *Staphylococcus epidermidis*, and *Pseudomonas aeruginosa* [3,5,6,7,8]. The incidence of the bacterial contamination of wounds not only prolongs the healing process, but it is also associated with patient discomfort, an extension of hospital stay, and generally in a significant increase in treatment cost [9]. Although the influx of neutrophils in the inflammation stage is aimed to keep the wound free of bacteria [4], the infections often occur in patients suffering from other diseases, which impede the standard progress of wound healing [2].

Many classes of drugs and drug combinations accelerate wound healing, protect peri-wound areas from infections, and reduce the risk of further infection complications. Various antimicrobial pharmacological approaches have so far been considered, including topical formulations with silver ions, antibiotics, or iodine-containing compounds [2]. In many other antimicrobial studies, new achievements of nanotechnology, materials chemistry, and medicinal chemistry have been implemented. These include applications of nanoparticles [5,10], biomaterials and wound dressings [11], peptides [12], polymers [13], natural products [14], bioengineered tissue substitutes [15], as well as systems for photodynamic therapy (PDT) [16,17] and photothermal therapy [18]. A great concern is associated with the factors that impede the healing of wounds [3]. The main issue is related to the contamination of damaged tissue. It can not only slow down the process of wound healing but also might spread the infection to other parts of the body. Therefore the use of additional antimicrobial treatment approaches is greatly required [2,5].

Both the red and the near-infrared light reach the deepest parts of tissues treated with photodynamic therapy. However, broader applications of PDT are limited due to the low penetration of light through tissues [19]. Therefore, photodynamic therapy against microorganisms, also called photodynamic antimicrobial chemotherapy (PACT), offers its full potential for treatment and healing of superficial infections [16,17]. The irradiated photosensitizer mediates the generation of reactive oxygen species, which can act destructively on the cells in the vicinity. PACT offers advantages over other antimicrobial therapies, mostly due to stopping the ability of pathogens to develop specific resistance mechanisms [20]. This treatment approach has been successfully studied on an array of bacterial, fungal, and viral strains, among them also multi-resistant ones, with various photosensitizers, such as porphyrinoids, curcumin, dipyrromethene dyes or nanoparticles [21,22,23,24,25].

**Figure 1:**
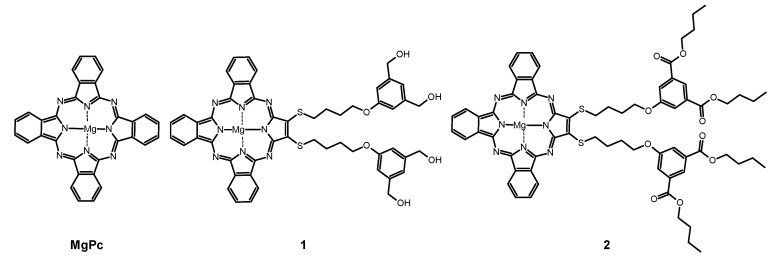
Chemical structures of studied macrocycles: MgPc, 1, and 2.

Herein we present the evaluation of the photokilling potential of tribenzoporphyrazines with bulky 4-(3,5-dihydroxymethylphenoxy)butylsulfanyl and 4-(3,5-dibutoxycarbonylphenoxy)butyl-sulfanyl moieties synthesized previously by our team [26] (Figure 1) against the wound bacteria. Studies were focused on the determination of the spectral properties, singlet oxygen formation evaluation, photostability evaluation as well as in vitro photocytotoxicity assessment against *Streptococcus pyogenes*, *Staphylococcus epidermidis*, and various methicillin-sensitive and -resistant *Staphylococcus aureus* strains.

## 2. Results and Discussion

### 2.1. Spectral Properties

**Figure 2:**
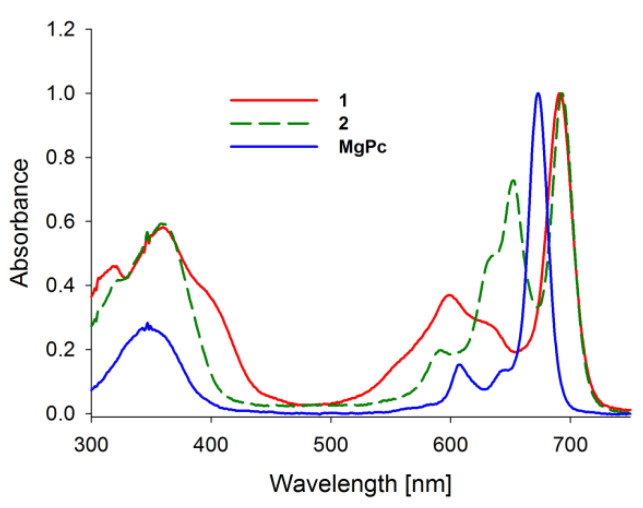
The UV-vis absorption spectra of studied macrocycles MgPc, 1, 2.

**Figure 3:**
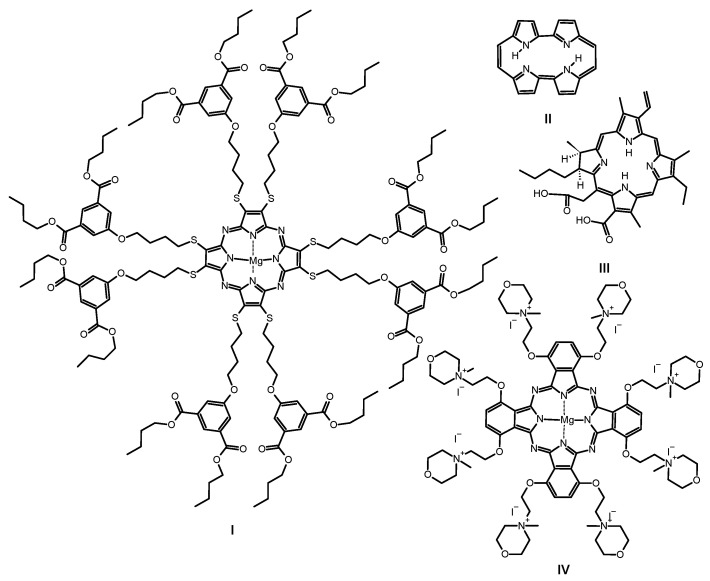
Chemical structures of previously studied macrocycles: sulfanylporphyrazine with isophthaloxybutyl substituents I, porphycene II, chlorin e6 III and phthalocyanine with quaternized morpholinethoxy substituents IV.

Absorption spectra of porphyrinoids consist of two characteristic bands (Figure 2). The first, known as the Soret band, is located in the near-ultraviolet and the blue region of the visible spectrum. The second, called the Q band, appears in the low-energetic, red region of the visible spectrum [27,28,29,30]. The Soret band is a result of π-π* electron transitions upon light quant absorption and is related to movement of electrons from HOMO (*a*_2*u*_) orbital to the LUMO (*e_g_*) orbital [31,32]. Herein studied compounds revealed the Soret band in the wavelength range of 250–450 nm with both maxima appearing at ca. 346 nm in solution. The Q bands were noted in the range of 550–750 nm as the result of π-π* electron transitions from HOMO to LUMO. For the reference MgPc, the profile of the phthalocyanine spectrum was typical for a symmetrical molecule with an intense sharp unsplit band maximum at 670 nm in DMF resulting from the electron transfer from *a_1u_* to degenerated *e_g_* orbital [33,34]. Interestingly, the split of the Q band in 1 and 2 is a consequence of a reduction in symmetry from D_4h_ observed for MgPc to C_2ν_ noted for both tribenzoporphyrazines. Also, a new component of the Q band was blue-shifted and revealed lower intensity than the Soret band. Moreover, flattening of the short wavelength component of the Q band in **1** in comparison with **2** was observed. It might be the result of the intramolecular coordination bond formed between an oxygen of the hydroxyl group present in the peripheral moiety and the central magnesium(II) ion. Recently, we reported that the appearance of intramolecular coordination is possible and that magnesium(II) ion creates relatively stable chelates [35]. Similarly to the report by Kobayashi [33], the long-wavelength component of the Q band of tribenzoporphyrazines 1 and 2 is ca. 30 nm red-shifted in comparison to the MgPc. It is related to the destabilization of molecular orbital in tribenzoporphyrazines. This phenomenon can also be responsible for lower intensities of the Q band [34]. Tribenzoporphyrazines 1 and 2 were compared to the previously reported porphyrazine I (Figure 3) [36]. An increase in the symmetry of I leads to the degeneration of *e_g_* orbital and is observed in the Q band as an unsplit sharp band. The molar absorption coefficients of MgPc (Table 1) indicate its high ability to absorb light. However, the decrease of symmetry and the introduction of sulfur atoms to the periphery of the molecule reduces logε to the value of 4.67 for 1. At ca. 500 nm, no band for 1 and 2 was observed as it had occurred in the case of I. The band at ca. 500 nm is a result of n-π* electron transfer from lone electron pair of the sulfur atom to the molecular orbital [37,38]. It seems that in the UV-vis spectra of studied tribenzoporphyrazines, the phenomenon of an n-π* electron transfer was negligible, whereas in the case of porphyrazine I, broadband with low intensity was observed [36]. Studied tribenzoporphyrazines presented a decreased ability of light absorption in comparison to MgPc (Table 1). Moreover, an increase in the Soret band intensities was noted in contrast to the Q bands for tribenzoporphyrazines (lgε[Q band]/lgε[Soret band] equal ca. 1.05, 1.04 and 1.11 for 1, 2 and MgPc, respectively).

**Figure 4:**
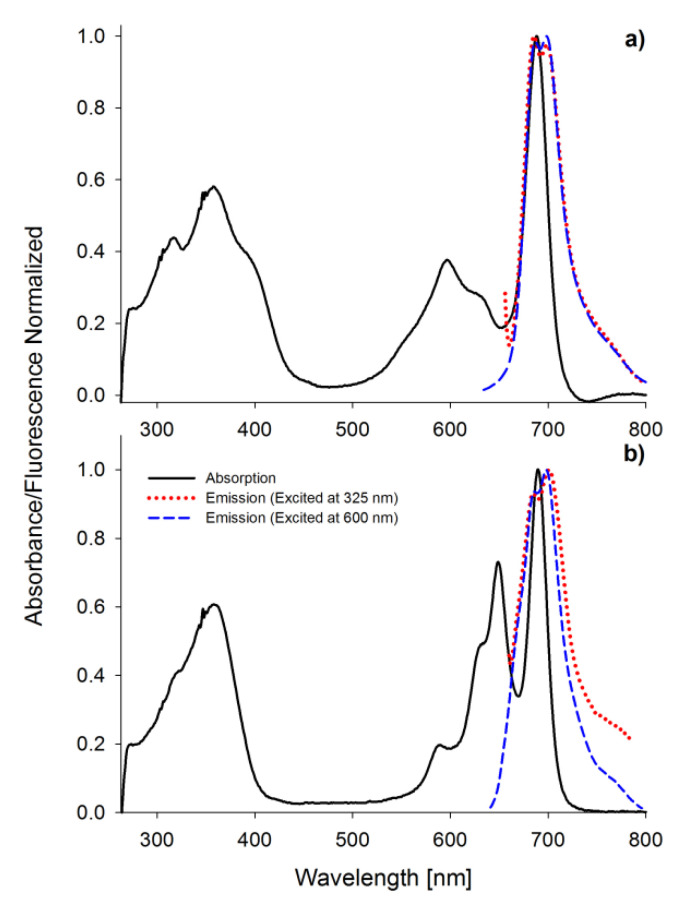
The UV-vis absorption and fluorescence spectra (excitation wavelength 325 and 600 nm) in DMF of 1 (**a**) and 2 (**b**).

Porphyrinoids easily emit fluorescence, and this phenomenon was also analyzed in the presented study. The researched tribenzoporphyrazines revealed only one emission band with the bathochromic shift in comparison to their absorption Q band (Figure 4). In contrast, sulfanylporphyrazine I, which is structurally related to 2, has shown dual emission. Dual emission is a result of two different excitation pathways. When the compound is excited with blue or ultraviolet light, electrons are transferred to the S_2_ state and rapidly come back to the ground state, simultaneously emitting light with the wavelength slightly higher than the absorption of the Soret band. If a compound is irradiated with red light (low energetic light), the electron transfer to the S_1_ state is observed, and the energy is lower than that for the transfer from the S_2_ state. The electrons returning to the ground state produce the red light emission at slightly higher wavelengths than Q band absorption [30,36]. The herein studied tribenzoporphyrazines 1 and 2, when excited with ultraviolet light, produced longwave emissions at the red region of the visible spectrum. It is caused by electron transformation and emission of light from the lowest excited state S_1_ upon irradiation with short-wavelength light. In the fluorescence spectra, minor band splitting was observed (Figure 4). A similar phenomenon has been reported by Harper and co-workers [39].

The highest quantum yield of fluorescence, equal to 0.230, was recorded for MgPc in DMF. This value was slightly higher than for ZnPc. Studied tribenzoporphyrazines 1 and 2 revealed fluorescence quantum yields values equal to 0.051 and 0.008, respectively. The introduction of the sulfur atom to 1 and 2 resulted in a decrease of the fluorescence, as reported before [40]. The lowered quantum yield values measured within studied sulfanyl tribenzoporphyrazines can be explained by the non-radiative electron transitions, the occurrence of which was reported by Ehrlich and co-workers [37]. Freyer and co-workers indicated that the extension of the macrocyclic ring from porphyrazine to tetrabenzoporphyrazine (phthalocyanine) increases fluorescence ability (Φ_FL_ of porphyrazine 0.17; Φ_FL_ of phthalocyanine 0.44) [41]. The same tendency was observed for I (0.005) [36], 2 (0.008) and MgPc (0.230).

### 2.2. Singlet Oxygen Formation

**Figure 5:**
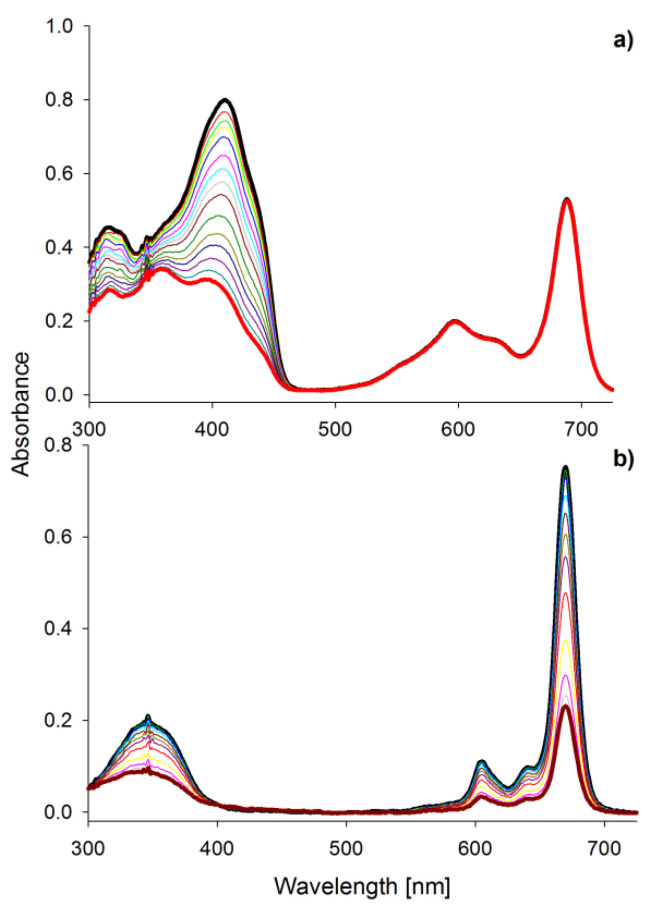
The UV-vis absorption spectra in DMF of (**a**) DPBF decomposition during irradiation of the mixture of 1 and DPBF within 540 s; (**b**) photobleaching of MgPc within 600 s.

Singlet oxygen is considered as the primary bacteria-killing agent in PACT [42]. Macrocycle 1 was found to be the best singlet oxygen generator within the studied tribenzoporphyrazines (Table 1, Figure 5a). Kobayashi reported that the splitting of the Q band impacts the Φ_∆_ value, and this feature can be used for the designing of macrocycles [33]. Symmetry is crucial for the comparison of 1, MgPc (Table 1), and previously reported compound I. In the case of I, no splitting in the Q band spectrum area was observed, and singlet oxygen quantum yield was equal 0.02 [36]. In comparison with MgPc and 1, for reference compound ZnPc, over 2-fold higher Φ_∆_ value was noted. It is caused by the widely described in the literature “heavy atom effect” [30]. The expansion of the porphyrazine leads to different Φ_∆_ values depending on the peripheral substituents. When benzene rings were fused with the magnesium(II) porphyrazine macrocycle, the value of singlet oxygen of 0.28 was noted. Interestingly, in the case of sulfanyl porphyrazine with eight isophthaloxybutyl substituents (I), the Φ_∆_ value reaches 0.02 [36]. The exchange of three pairs of sulfanyl substituents of porphyrazine I for three fused benzene rings in the tribenzoporphyrazine 2 resulted in the increase of Φ_∆_ value up to 0.05. Most of the sulfanyl porphyrazines studied in our group presented low singlet oxygen generation ability (Φ_∆_ below 0.1), which could be linked with the impact of the sulfur atom on the macrocycle electronic structure [26,40,43,44]. Differently, compound 1 as a member of sulfanyl tribenzoporphyrazines reached Φ_∆_ up to 0.27 in DMSO. This tendency can be explained by the possibility to form dimers by I and aggregates by tribenzoporphyrazines [36,45]. Higher singlet oxygen generation yields of 1 might be explained by the formation of the intramolecular complex between magnesium(II) ion and the oxygen of the hydroxyl group present in the peripheral substituents, which effectively block the π-π stacking interaction between molecules.

### 2.3. Photostability

Irradiation of porphyrinoids leads to the formation of low weight, colorless compounds, or other macrocyclic molecules, which is known as photobleaching or phototransformation, respectively [50]. Studied compounds irradiated with visible light underwent photobleaching processes as an irreversible decrease in the intensity of their absorption bands was observed (Figure 5b). The most unstable compound was MgPc in DMF with the photodecomposition quantum yield Φ_P_ = 32.7 × 10^−5^, whereas the most photostable were MgPc and 2 dissolved in DMSO with the Φ_P_ 4.77 × 10^−6^ and 4.84 × 10^−6^ respectively. This difference can be explained by the nature of applied solvent. It has previously been reported that DMSO and oxygen atoms easily coordinate with the magnesium(II) ion located in the center of the macrocyclic ring [35]. Kuznetsova and Kaliya associated coordination of DMSO to central metal ion to the lowest photodecomposition quantum yields [51]. Macrocycle 1 presented high Φ_P_ in DMSO, which could be linked with relatively high singlet oxygen quantum yield of this compound (Table 1). Herein studied porphyrinoids, except MgPc, reveal photostability quantum yields at the 10^−5^ and 10^−6^ level, which allows for classifying them as stable photosensitizers. Dilber and co-workers have divided PSs into the stable and unstable group considering Φ_P_ values [52]. Compounds with Φ_P_ ca. 10^−3^ were categorized as labile, whereas those with Φ_P_ at ca. 10^−6^ as stable. High lability of magnesium(II) phthalocyanine derivative was described by Łapok and co-workers as well [53]. Photoreactivity of magnesium(II) porphyrinoids, i.e., porphyrins, chlorophylls, and phthalocyanines, was reported by Sergeeva and Senge [54]. They explained this feature with the susceptibility of magnesium(II) macrocycles to the demetallation process.

### 2.4. Photodynamic Activity against Bacteria

The antibacterial photodynamic activity was assessed against Gram-positive bacteria derived from wound infections as nearly three-quarters of nosocomial infections have been related to surgical wounds [6]. Nowadays, the challenge posed for medical care is also in the treatment of chronic wounds, which consume 2–4% of the medical system finance of western countries [7]. The main Gram-positive bacteria in the infected wound development are *Staphylococcus aureus*, including *MRSA* and *Streptococcus pyogenes* [6,7,8].

The researched macrocycles were encapsulated in liposome vehicles as they present a hydrophobic nature. Liposomes were prepared using a thin-film hydration method. The positive charge, important to sufficient bacteria photoinactivation, was provided by a liposome ingredient—chloride salt of DOTAP. The size of the liposomes corresponded to earlier prepared formulations [55]. The average diameter of the obtained liposomes was measured at 301 nm, 188 nm, and 119 nm for 1, 2, and MgPc, respectively (Table 2). Liposome vehicles were used for solubilization of lipophilic photosensitizers enabling to keep them in the monomeric, non-aggregated forms, which is necessary for high photodynamic activity [56].

It should be underlined that all the studied compounds revealed high antibacterial activity with log reduction above three log—the FDA’s arbitrary threshold for bactericidal substances [42].

Statistically significant bacteria reduction for 1 was found at the concentration of 10^−5^ M (*p* = 0.02), for 2 at the concentration of 10^−4^ M (*p* = 0.04) and 10^−5^ M (*p* = 0.02) and for MgPc at 10^−4^ M and 10^−5^ M (*p* = 0.02).

The primary wound pathogen is *S. aureus*, responsible for the majority of human infections from mild to life-threatening, including bloodstream, skin, mucosa, soft tissues, and many other infections [57]. Among the studied macrocycles, tribenzoporphyrazine 1 presented the highest *S. aureus* killing rate of over 5.9 log at the concentration of 10^−5^ M, and under irradiation with a relatively low light dose of 30 J/cm^2^. Similar results of *S. aureus* photodynamic inactivation was reported before [56]. Interestingly, at higher concentrations, studied compounds revealed lower photodynamic bacterial growth reduction potential. This can be related to the π-π stacking phenomenon and aggregation. The obtained killing rate for 1 at 10^−5^ M concentration can be considered as total bacteria eradication. The same *S. aureus* photokilling level at similar dosimetry was reported by Masiera and co-workers, who noted 6 log (complete eradication) reduction in bacterial growth for unsubstituted porphycene (II, Figure 3) loaded into micelles at the concentration of 7 × 10^−6^ M [58]. Another macrocycle, phthalocyanine IV (Figure 3), presented higher activity at 10^−4^ M than at 10^−5^ M [59], which can be linked to a decreased tendency to form aggregates as the result of electrostatic repulsion between single molecules. The other main wound bacterium is methicillin-resistant *Staphylococcus aureus* (MRSA). This *S. aureus* strain appeared in the 1960s and still causes severe medical treatment issues up to the present day. This bacterial strain is still developing resistance to all known antibiotics. It is estimated that from 25 to 50% *S. aureus* hospital infections are in fact caused by the MRSA strains. Therefore, some institutions regard it as a top-priority drug-resistant microbe [57,60]. MRSA is also susceptible to the evaluated macrocycles. At 10^−4^ M concentration, tribenzoporphyrazines 1 and 2 presented no activity, whereas, at a 10-fold lower dose, a significant increase in photodynamic activity of up to 5.1 log was observed.

The third most common wound bacterium is *Streptococcus pyogenes*, which mainly causes mild superficial infections i.e., impetigo. However, rare but life-threatening incidents of superficial (skin, mucosa) infections are on the rise, including necrotizing fasciitis and Streptococcal toxic shock syndrome resulting in high mortality [8,61,62]. For the photodynamic treatment of *S. pyogenes*, a similar pattern as for MRSA was observed, but the log reduction of growth values was slightly lower (Table 3). The photodynamic elimination was examined not only against main wound microbes but also towards *S. epidermidis*—a skin microbiota species. This pathogen is especially dangerous for neonates, as it might cause sepsis, often leading to death [63,64]. Studied macrocycles revealed *S. epidermidis* photokilling rate at log values similar to those measured for *S. aureus*, up to over 5.7 log (10^−5^ M). Interestingly, macrocycle II loaded into pluronic micelles has reduced *S. epidermidis* colonies at the dose of 7 × 10^−6^ M and the light dose of only 6 J/cm^2^ [58]. A well-known photosensitizer, chlorin e6 (III, Figure 3), presented 6 log reduction in *S. epidermidis* growth when it was delivered at the same concentration (10^−5^ M), but using a 2-fold higher light dose (55 J/cm^2^) [65], in comparison to 1 and 2. Recently reported magnesium(II) phthalocyanine bearing eight positive charges at the periphery (IV) presented a high growth reduction of *S. epidermidis* of 5.42 log at the concentration of only 10^−4^ M. Interestingly, when IV was tested at 10^−5^ M concentration, bacteria reduction dropped to 2.48 log [59], which is lower than the FDA recommendation for bactericidal compounds [42]. Tribenzoporphyrazines 1 and 2 at 10^−4^ M showed lower photoactivity than at 10^−5^ M concentration. This might suggest that 10^−5^ M concentration is the limit for providing a monomeric form of the photosensitizers in POPC:DOTAP liposomes. Taking into account the results reported by Dlugaszewska and co-workers [59], and the outcomes obtained in this study, positively charged liposomes loaded with neutral photosensitizers provide at least the same levels of Gram-positive bacteria reduction when compared to positively charged photosensitizer molecules. The photocytotoxicity of tribenzoporphyrazines 1 and 2 against cancer cells was evaluated before. It was reported that among the studied liposomal formulations loaded with 1 and 2, POPC:DOTAP liposomes presented the lowest IC_50_ values against oral carcinoma cell lines (CAL-27, HSC-3) [45,55]. A simple topical administration route and an easy light delivery create perspectives for the effective treatment of bacterial infections associated with oral cancers in one medical procedure.

## 3. Materials and Methods

### 3.1. Materials

Studied compounds: 22,23-bis{4-[3,5-di(hydroxymethyl)phenoxy]butylsulfanyl}tribenzo[b,g,l]-porphyrazinato magnesium(II) (**1**) and 22,23-Bis [4-(3,5-dibutoxycarbonylphenoxy)butylsulfanyl]tri-benzo[b,g,l]porphyrazinato magnesium(II) (**2**) (Figure 1) were prepared according to earlier presented procedures [26]. The unsubstituted magnesium(II) phthalocyanine (MgPc) was purchased in Aldrich (Saint Louis, MO, USA). POPC (1-palmitoyl-2-oleoyl-sn-glycero-3-phosphocholine) and DOTAP (*N*-[1-(2,3-dioleoyloxy)propyl]-*N*,*N*,*N*-trimethylammonium chloride) were purchased from Avanti Polar Lipids Inc. (Alabaster, AL, USA).

### 3.2. Spectral Properties

Absorption spectra and emission spectra of the studied compounds were recorded on a Shimadzu UV-160 spectrophotometer and a JASCO 6200 spectrofluorometer, respectively, in *N*,*N*-dimethylformamide (DMF) and dimethylsulfoxide (DMSO) solutions at ambient temperature [47,66,67].

### 3.3. Singlet Oxygen Generation

Singlet oxygen generation quantum yields were determined in DMF and DMSO solutions under the aerobic atmosphere at ambient temperature. Experiments were performed with a comparative method previously described [67]. DPBF (1,3-diphenylisobenzofuran, Aldrich, Saint Louis, MO, USA)) was used as a singlet oxygen chemical trap. The obtained kinetic parameters of DPBF decomposition were referred to unsubstituted zinc(II) phthalocyanine (Aldrich, Saint Louis, MO, USA)) with known singlet oxygen generation quantum yield values. The mixture of DPBF and studied macrocycle was irradiated with light at the Q band maximum. The preferred wavelength of light was separated with the monochromator (M250/1200/U with 2 nm/mm dispersion, Dk = 4 nm, Optel, Opole, Poland). A high-pressure xenon lamp with a broad emission spectrum (150 W, Optel, Opole, Poland) was used as a light source. In the determined time intervals, the UV-vis absorption spectra were recorded with a Shimadzu UV-160 spectrophotometer.

### 3.4. Photostability Determination

The photodegradation quantum yields were determined in DMF and DMSO under the aerobic atmosphere at ambient temperature following the method previously applied [68,69,70,71]. The high-pressure xenon lamp was used as a light source (150 W, Optel). The high energy light below 450 nm was cut off with the filter. Spectra were recorded at certain time intervals with a Shimadzu UV-160 spectrophotometer.

### 3.5. Photodynamic Activity against Bacteria

#### 3.5.1. Liposomes Preparation and Determination

Liposomes were prepared with the thin-film hydration method followed by extrusion. POPC and DOTAP were mixed in a molar ratio of 8:2. Next, chloroform solutions containing 1, 2, and MgPc (1 mg/mL) were diluted to the final concentration of 200 µM in the vesicle. Then, the obtained mixture was evaporated under reduced pressure leading to a lipid film. Further, phosphate-buffered saline (PBS) was added and vortexed for 5 min. The unification process was performed by the extrusion of water mixture through the polycarbonate filter (200 nm). The size of the obtained vesicles was measured with NanoSight LM10 (Malvern Panalytical, Malvern, UK).

#### 3.5.2. Bacterial Strains and Culture Conditions

The following wound Gram-positive bacteria were chosen: the clinical strains of methicillin-resistant *Staphylococcus aureus* (MRSA) and *Streptococcus pyogenes*, as well as ATCC collection strains of *Staphylococcus aureus* (ATCC 25923) and *Staphylococcus epidermidis* (ATCC 49134). The clinical isolates were obtained from bacterial strains collection of the Department of Genetics and Pharmaceutical Microbiology, Poznan University of Medical Sciences. The bacteria were cultured aerobically in Brain Heart Infusion (BHI) broth (Becton Dickinson, Franklin Lakes, NJ, USA) at 36 °C ± 1 °C for 20 h. Next, the bacterial cultures were centrifuged at 3000 rpm for 15 min, the supernatant was discarded, and the cells pellet was re-suspended and diluted in 10 mM PBS (pH = 7.0) to a final concentration of ca. 10^7^ Colony Forming Units (CFU)/mL.

#### 3.5.3. Photodynamic Activity

The photodynamic effect of photosensitizers (PSs) was evaluated by measuring the reduction in viability of microbial cells. Therefore, aliquots of the bacterial suspensions were placed in the 96-wells microplate. Next, the suspension of PS-loaded liposomes was added at appropriate volumes to achieve desired concentrations in the wells. The control samples were also prepared. The cultures were kept in the dark for 20 min (incubation time). Two sets of experiments were prepared. After incubation time one set was irradiated by the LED MultiChip Emitter consisting of 60 high-efficiency AlGaAs diode chips (Roithner LaserTechnik GmbH, Vienna, Austria) with maximum wavelength 690 nm, corresponding to the Q band of the PSs, at a fixed total light dose 30 J/cm^2^ (light phase). The dark phase was also evaluated. After incubation time (20 min), both light and dark phase cultures were transferred to the Tryptic Soy Agar (TSA, OXOID, Basingstoke, UK) plates (*S. aureus*, MRSA, *S. epidermidis*) and Columbia Agar with sheep blood (CBA, OXOID, UK) plates (*S. pyogenes*). After overnight incubation at 36 °C ± 1 °C (*S. pyogenes* was cultured in a CO_2_ enriched atmosphere), the CFUs were counted, and the number of viable microorganisms was calculated (number of CFU/mL). Next, the log reduction of living bacteria in each sample was determined. Experiments were performed in triplicates.

#### 3.5.4. Statistical Analysis

The statistical analyses were performed using the STATISTICA software, v.13.0. The data for CFU/mL were converted to the logarithmic form. Pairwise comparisons were performed using the unpaired Student’s t, and U Mann-Whitney tests to determine whether a signifificant reduction in colony-forming units occurred as comparing irradiated to the non-irradiated samples. Data were analyzed for statistical signifificance with the Dunnett’s Multiple Comparison test. A probability value (*p*) of <0.05 was considered as signifificantly difffferent.

## 4. Conclusions

The optical properties and photocytotoxicities of two magnesium(II) tribenzoporphyrazines, bearing 4-(3,5-dihydroxymethylphenoxy)butylsulfanyl or 4-(3,5-dibutoxycarbonylphenoxy)butyl-sulfanyl substituents at the periphery, were studied. In the UV-vis absorption spectra, compounds in solutions showed the Soret bands in the wavelength range of 250–450 nm with the maxima at ca. 346 nm and the Q bands in the range of 550–750 nm. Interestingly, the spectra of both tribenzoporphyrazines were split in the Q band regions, and both molecules presented a decreased ability of light absorption in comparison to MgPc. Moreover, an increase of Soret bands intensity in comparison to Q bands for tribenzoporphyrazines was observed in comparison to MgPc (lgε[Q band]/lgε[Soret band] equal ca. 1.05, 1.04 and 1.11 for 1, 2 and MgPc respectively). Macrocycles 1 and 2 showed only one emission band shifted bathochromically when compared with the absorption Q band. In the fluorescence spectra, minor band splitting was observed. The highest quantum yield of fluorescence was recorded for MgPc in DMF, equal 0.28. The studied tribenzoporphyrazines presented fluorescence quantum yield values of 0.051 and 0.008 for 1 and 2, respectively. Within the studied tribenzoporphyrazines, macrocycle **1** was found to be the best singlet oxygen generator. The reference ZnPc presented over 2-fold higher Φ_∆_ value than MgPc and 1. Noteworthy is the fact that macrocycle 1 as a member of sulfanyl tribenzoporphyrazines reached Φ_∆_ up to 0.27 in DMSO in comparison to other sulfanyl porphyrazines for which the Φ_∆_ usually does not exceed 0.1. The studied compounds underwent photobleaching processes when irradiated with visible light, and the ratios of the processes were linked to the used solvent. The most unstable compound was MgPc in DMF with the photodecomposition quantum yield Φ_P_ equal 32.7 × 10^−5^, whereas the most photostable were MgPc and 2 with Φ_P_ equal 4.77 × 10^−6^ and 4.84 × 10^−6^ in DMSO solutions, respectively. Contrarily, 1 in DMSO presented high Φ_P_, which could be related to its relatively high singlet oxygen quantum yield in this solvent. Both tribenzoporphyrazines revealed photostability quantum yield values spanning between 10^−5^ and 10^−6^, which allows to classify them as stable photosensitizers.

Antibacterial photodynamic activity was assessed against Gram-positive bacteria derived from the wound infections *Staphylococcus aureus*, including MRSA and *Streptococcus pyogenes*. Due to the hydrophobic character of the studied compounds, they were encapsulated into liposome vehicles. Liposomes were obtained with an average diameter equal 301 nm, 188 nm, and 119 nm for 1, 2, and MgPc, respectively. Among the studied macrocycles, tribenzoporphyrazine 1 revealed the highest *S. aureus* killing rate of over 5.9 log at the concentration of 10^−5^ M. Its activity was observed under irradiation with a relatively low light dose of 30 J/cm^2^. When evaluated against MRSA, tribenzoporphyrazines 1 and 2 were not active at the concentration of 10^−4^ M, whereas, at a 10-fold lower dose, their significant increase in photocytotoxicity, of up to 5.1 log reduction was noted. Similar regularity was observed for *S. pyogenes*, but the log reduction values were somewhat lower. Tested macrocycles also revealed photokilling of *S. epidermidis*, naturally occurring on skin, at log values similar to those observed for *S. aureus* up to over 5.7 (10^−5^ M). According to the herein presented data, positively charged liposomes loaded with non-charged photosensitizers provide similar efficacy for Gram-positive bacteria photokilling potential as positively charged molecules of photosensitizers.

## Figures and Tables

**Table 1:** Fluorescence, photodegradation, singlet oxygen formation quantum yields and log of the molar absorption coefficient of 1, 2 and MgPc.

Compound	Solvent	Φ_FL_	10^6^Φ_P_	Φ_Δ_	logε[nm]	
1	DMF	0.051	86.40	0.20 [26]	4.49[346]	4.67[688]
	DMSO	0.029	37.60	0.27	4.45[346]	4.67[691]
2	DMF	0.008	48.40	0.05 [26]	4.69[346]	4.83[690]
	DMSO	0.002	4.84	0.08	4.65[346]	4.84[693]
MgPc	DMF	0.230 [46]	327 [46]	0.28 [46]	4.91[346]	5.36[670]
	DMSO	0.181	4.77	0.14	4.80[346]	5.33[673]
ZnPc	DMF	0.200 [47]	10.2 [48]	0.56 [49]	-	
	DMSO	0.170 [47]	3.5 [48]	0.67 [49]		

**Table 2:** Liposome particle size analysis.

Compound	Mean Diameter [nm]	Dv10 [nm]	Dv50 [nm]	Dv90 [nm]
1	301 ± 3	126	217	629
2	188 ± 23	71	147	346
MgPc	119 ± 4	65	115	171

**Table 3:** Photodynamic reduction of the bacterial growth by the studied compounds.

Compound	1	2	MgPc
Concentration [M]	log reduction in bacterial growth		
*Staphylococcus aureus*			
10^−4^	3.5 ± 0.3	3.6 ± 0.2	4.9 ± 0.3
10^−5^	>5.9 ± 0.0	4.3 ± 0.1	4.6 ± 0.2
*MRSA*			
10^−4^	no activity	0.7 ± 0.1	>5.7 ± 0.1
10^−5^	5.1 ± 0.1	3.8 ± 0.0	>5.7 ± 0.1
*Staphylococcus epidermidis*			
10^−4^	3.3 ± 0.2	3.6 ± 0.2	>6.0 ± 0.0
10^−5^	>5.7 ± 0.2	>5.7 ± 0.3	>5.7 ± 0.4
*Streptococcus pyogenes*			
10^−4^	0.5 ± 0.3	1.7 ± 0.2	>4.7 ± 0.3
10^−5^	>4.7 ± 0.3	>4.7 ± 0.3	>4.7 ± 0.3
